# The MultiFurcating Neighbor-Joining Algorithm for Reconstructing Polytomic Phylogenetic Trees

**DOI:** 10.1007/s00239-023-10134-z

**Published:** 2023-10-21

**Authors:** Alberto Fernández, Natàlia Segura-Alabart, Francesc Serratosa

**Affiliations:** 1 Departament d’Enginyeria Química, Universitat Rovira i Virgili, Tarragona, Spain; 2 Departament d’Enginyeria Informàtica i Matemàtiques, Universitat Rovira i Virgili, Tarragona, Spain

**Keywords:** Distance-based methods, Neighbor-joining, Polytomy, Ties in proximity

## Abstract

Results from phylogenetic analyses that study the evolution of species according to their biological characteristics are frequently structured as phylogenetic trees. One of the most widely used methods for reconstructing them is the distance-based method known as the neighbor-joining (NJ) algorithm. It is known that the NJ algorithm can produce different phylogenetic trees depending on the order of the taxa in the input matrix of evolutionary distances, because the method only yields bifurcating branches or dichotomies. According to this, results and conclusions published in articles that only calculate one of the possible dichotomic phylogenetic trees are somehow biased. We have generalized the formulas used in the NJ algorithm to cope with Multifurcating branches or polytomies, and we have called this new variant of the method the multifurcating neighbor-joining (MFNJ) algorithm. Instead of the dichotomic phylogenetic trees reconstructed by the NJ algorithm, the MFNJ algorithm produces polytomic phylogenetic trees. The main advantage of using the MFNJ algorithm is that only one phylogenetic tree can be obtained, which makes the experimental section of any study completely reproducible and unbiased to external issues such as the input order of taxa.

## Introduction

The neighbor-joining (NJ) algorithm is a well-known distance-based method for reconstructing phylogenetic trees, introduced by Saitou and Nei (1987). It is an agglomerative or bottom-up method that forms a phylogenetic tree grouping pairs of taxa into nodes in a greedy manner. Since its publication, the NJ algorithm has become the most widely used method for building phylogenetic trees from distances (Gascuel and Steel 2006).

Any taxon in a leaf and any internal node of a phylogenetic tree is called an operational taxonomic unit (OTU). It is long known that more than one phylogenetic tree can be obtained when there are identical sums of branch lengths between different pairs of OTUs, at any iteration of the agglomerative process guided by the NJ algorithm. This characteristic of the algorithm is known as the ties in proximity problem (Backeljau et al. 1996). When different phylogenetic trees are possible, the reproducibility of the results is complicated and generally biased towards just one of the possible solutions. Thus, any conclusion acquired from a single phylogenetic tree is partial and, therefore, questionable (Segura-Alabart et al. 2022).

Over the last years, the scientific community has developed several alternative versions of the NJ algorithm. To name just a few, there are algorithms that use heuristics to reduce their running time, making them suitable for large-scale applications: QuickTree (Howe et al. 2002), QuickJoin (Mailund and Pedersen 2004), relaxed neighbor joining (Evans et al. 2006), and fast neighbor joining (Elias and Lagergren 2009). Some algorithms try to recover the minimum evolution tree keeping track of several partial solutions along the execution of the algorithm and, thus, exploring a greater part of the tree space: generalized neighbor joining (Pearson et al. 1999), neighbor-joining maximum likelihood (Ota and Li 2000), and multi-neighbor joining (Silva et al. 2005). And other possibilities include BIONJ (Gascuel 1997) and weighted neighbor joining (Bruno et al. 2000), which consider differently long genetic distances than short ones, and MJOIN (Levy et al. 2006), which uses estimates of phylogenetic diversity rather than pairwise distances in the tree.

However, none of the above alternatives to the NJ algorithm addresses the ties in proximity problem. This problem arises because the NJ algorithm creates internal nodes that are always dichotomies. An internal node of a phylogenetic tree is a dichotomy when the tree is rooted and the node is linked to two child subtrees, or when the tree is unrooted and three branches are connected to the node. If more branches are connected to an internal node, then we have a polytomy.

Solutions based on the conversion of multiple bifurcating trees into a single multifurcating tree using any consensus method would be computationally inefficient because an exhaustive search for all possible bifurcating trees would be needed. In addition, general users are unaware of the ties in proximity problem, and they do not run the NJ method several times changing the input order of taxa to check whether different phylogenetic trees are obtained.

In order to allow for polytomies, one could use phylogenetic networks that, in spite of no longer trees, can present a unique network for a matrix of evolutionary distances (Bryant and Moulton 2004). Another possibility could be to modify the NJ algorithm accordingly. As a matter of fact, the NJ algorithm itself is based on the simultaneous partitioning method by Saitou (1986), which considers all possible partitions of *N* OTUs into two clusters with *m* and *n* OTUs respectively ($m + n = N$; $m,n \ge 2$), and selects the best one. Unfortunately, considering all possible partitions into two clusters has the problem of combinatorial explosion (Saitou 2018).

We introduce here a generalization of the formulas used in the NJ algorithm so that they can create internal nodes that are polytomies. We have called the method that uses these formulas the multifurcating neighbor-joining (MFNJ) algorithm, which always returns a unique phylogenetic tree independently of the order of the taxa in the input matrix of evolutionary distances. That is, the algorithm presented here is capable of grouping any number of OTUs at the same time, and therefore, it is not affected by the ties in proximity problem. Besides, when there are no ties, the MFNJ algorithm gives the same results as the NJ algorithm.

## Methods

In this section, we first review the formulas used in the NJ algorithm, and then we explain our proposal to generalize them.

### Neighbor-Joining

**Fig. 1:**
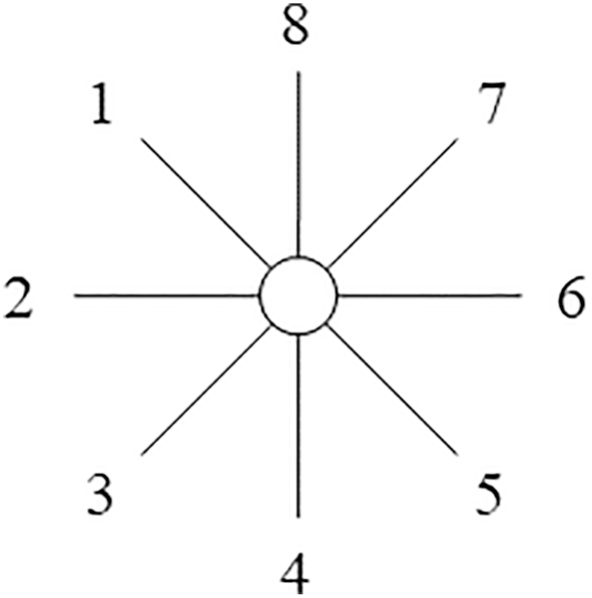
A starlike tree with no hierarchical structure

The NJ algorithm builds a phylogenetic tree from a matrix of evolutionary distances, $D_{ij}$, between each pair of taxa *i*, *j* under study. The whole set of taxa is taken as the starting set of OTUs, and they are initially arranged in a starlike tree as in Fig. 1, assuming that there is no clustering of OTUs. In each iteration of the algorithm, the values $S_{ij}$ are calculated for each pair of OTUs *i*, *j* as follows:

1 $$\begin{aligned} S_{ij} = (N-2) D_{ij} - R_{i} - R_{j}, \end{aligned}$$

where *N* is the current number of OTUs, and $R_{i}$ is the sum of distances between OTU *i* and all the other OTUs:

2 $$\begin{aligned} R_{i} = \sum _{k} D_{ik}. \end{aligned}$$

Note that Equation (1) is the one in Studier and Keppler (1988), and minimizing it is equivalent to minimizing the sum of branch lengths of Saitou and Nei (1987) (Gascuel 1994).

**Fig. 2:**
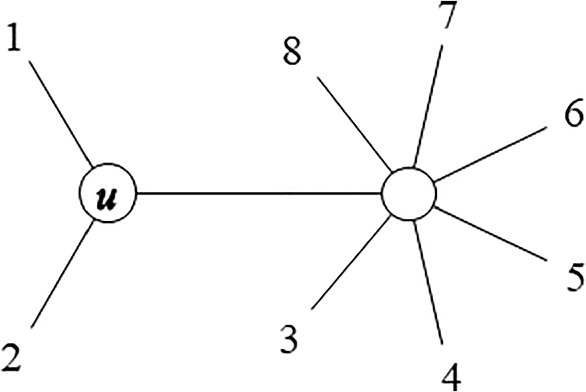
A tree with OTUs $I=\{1,2\}$ joined to new node *u*

A pair of OTUs for which $S_{ij}$ is the smallest is selected. Let $I=\{i_{1},i_{2}\}$ be a pair of selected OTUs that minimize $S_{ij}$. Then, $i_{1}$ and $i_{2}$ are clustered together generating a new internal node *u* (refer Fig. 2), and the distance between the new node *u* and any other OTU $k \ne i_{1},i_{2}$ is calculated as follows:

3 $$\begin{aligned} D_{uk} = \frac{D_{i_{1}k} + D_{i_{2}k}}{2} - \frac{D_{i_{1}i_{2}}}{2}. \end{aligned}$$

Again, Equation (3) is the one in Studier and Keppler (1988), not the one in Saitou and Nei (1987), although they are equivalent and both of them reconstruct the same tree (Gascuel 1994).

Finally, the length of the new branch linking $i_{1}$ and *u* is calculated as follows:

4 $$\begin{aligned} L_{i_{1}u} = \frac{D_{i_{1}i_{2}}}{2} + \frac{R_{i_{1}I^{\complement }}}{N-2} - \frac{R_{II^{\complement }}}{2(N-2)}, \end{aligned}$$

where $I^{\complement }$ is the complement of *I*, $R_{iI^{\complement }}$ is the sum of distances between an OTU $i \in I$ and all the other OTUs $k \notin I$:

5 $$\begin{aligned} R_{iI^{\complement }} = \sum _{k \notin I} D_{ik}, \end{aligned}$$

and $R_{II^{\complement }}$ is the sum of distances between all the OTUs $i \in I$ and all the other OTUs $k \notin I$:

6 $$\begin{aligned} R_{II^{\complement }} = \sum _{i \in I} \sum _{k \notin I} D_{ik}. \end{aligned}$$

$L_{i_{2}u}$ can be obtained in the same way or simply subtracting $L_{i_{1}u}$ from $D_{i_{1}i_{2}}$.

In each iteration, the two selected OTUs, $i_{1}$ and $i_{2}$, are removed from the distance matrix, *D*, and a new internal node *u* is added. The procedure ends when the current number of OTUs is equal to three, and there is only one possible unrooted tree. The branch length for each one of the last three OTUs, $i_{1}$, $i_{2}$ and $i_{3}$, is calculated as follows:

7 $$\begin{aligned} L_{i_{1}u} = \frac{D_{i_{1}i_{2}} + D_{i_{1}i_{3}} - D_{i_{2}i_{3}}}{2}. \end{aligned}$$

Note that in the NJ algorithm, if more than one pair of OTUs have the smallest $S_{ij}$, only one pair can be selected. To avoid any arbitrary decision, we propose a generalization of the method capable to cope with the selection of more than one pair of OTUs.

### MultiFurcating Neighbor-Joining

The method we propose, the MFNJ algorithm, is a generalization of the NJ algorithm. Both algorithms use Equation (1) to compute $S_{ij}$ in the same way, where the two algorithms diverge is in the procedure for joining OTUs. Suppose that, in a specific iteration, two pairs of OTUs, $i_{1},i_{2}$ and $i_{2},i_{3}$, have the smallest $S_{ij}$; that is, $S_{i_{1}i_{2}}=S_{i_{2}i_{3}}=S_{\min }$. In this case, the NJ algorithm can only join one of these pairs of OTUs, $i_{1},i_{2}$ or $i_{2},i_{3}$, to generate a new internal node *u*, which pair is selected has consequences for the next steps of the NJ algorithm. In the MFNJ algorithm, given that both pairs of OTUs, $i_{1},i_{2}$ and $i_{2},i_{3}$, have $i_{2}$ in common, we propose to generate a new internal node *u* joining the set of three OTUs $I=\{i_{1},i_{2},i_{3}\}$.

#### Distance Between an Internal Node and an OTU

More generally, let $I=\{i_{1},i_{2},\ldots ,i_{P}\}$ be a set of OTUs to be clustered together generating a new internal node *u*. The distance between any OTU $i \in I$ and any other OTU $k \notin I$ can be separated in two parts (refer Fig. 2):

8 $$\begin{aligned} D_{ik} = L_{iu} + D_{uk}. \end{aligned}$$

Taking this equality for all the OTUs $i \in I$, the distance between the new node *u* and any OTU $k \notin I$ can be averaged as follows:

9 $$\begin{aligned} D_{uk} = \frac{1}{|I|} \sum _{i \in I} \left( D_{ik} - L_{iu} \right) , \end{aligned}$$

where |*I*| is the number of OTUs to be joined to the internal node *u*. Now, using the equality that Saitou and Nei (1987) gave for the sum of branch lengths of a starlike tree with central node *u*:

10 $$\begin{aligned} \sum _{i \in I} L_{iu} = \frac{R_{II}}{|I|-1}, \end{aligned}$$

where $R_{II}$ is the sum of distances between all the OTUs in *I*:

11 $$\begin{aligned} R_{II} = \sum _{i \in I} \sum _{\begin{array}{c} i' \in I \\ i'>i \end{array}} D_{ii'}, \end{aligned}$$

we finally propose to generalize Equation (3) for the calculation of the distance $D_{uk}$ between the new node *u* and any other OTU $k \notin I$ as follows:

12 $$\begin{aligned} D_{uk} = \frac{R_{Ik}}{|I|} - \frac{R_{II}}{|I|(|I|-1)}, \end{aligned}$$

where $R_{Ik}$ is the sum of distances between all the OTUs in *I* and OTU $k \notin I$:

13 $$\begin{aligned} R_{Ik} = \sum _{i \in I} D_{ik}. \end{aligned}$$

#### Distance Between Two Internal Nodes

**Fig. 3:**
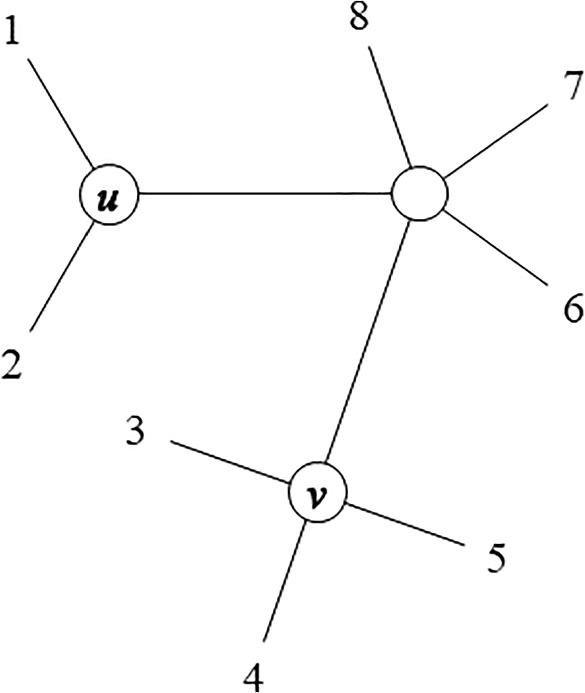
A tree with OTUs $I=\{1,2\}$ joined to a new node *u*, and OTUs $J=\{3,4,5\}$ joined to another new node *v*, during the same iteration of the algorithm

As a matter of fact, there may be cases where more than one set of OTUs can be clustered during the same iteration of the algorithm, being these sets of OTUs disjoint sets. In these cases, when there are two new internal nodes *u* and *v* joining two disjoint sets of OTUs $I=\{i_{1},i_{2},\ldots ,i_{P}\}$ and $J=\{j_{1},j_{2},\ldots ,j_{Q}\}$, respectively, the distance between any OTU $i \in I$ and any other OTU $j \in J$ can be separated in three parts (refer Fig. 3):

14 $$\begin{aligned} D_{ij} = L_{iu} + D_{uv} + L_{jv}. \end{aligned}$$

Taking this equality for all the OTUs $i \in I$ and $j \in J$, the distance between the new nodes *u* and *v* can be averaged as follows:

15 $$\begin{aligned} D_{uv} = \frac{1}{|I||J|} \sum _{i \in I} \sum _{j \in J} \left( D_{ij} - L_{iu} - L_{jv} \right) , \end{aligned}$$

which, using Equation (10), can be expressed as follows:

16 $$\begin{aligned} D_{uv} = \frac{R_{IJ}}{|I||J|} - \frac{R_{II}}{|I|(|I|-1)} - \frac{R_{JJ}}{|J|(|J|-1)}, \end{aligned}$$

where $R_{IJ}$ is the sum of distances between pairs of OTUs in *I* and *J*:

17 $$\begin{aligned} R_{IJ} = \sum _{i \in I} \sum _{j \in J} D_{ij}, \end{aligned}$$

and $R_{II}$ and $R_{JJ}$ are calculated using Equation (11).

#### Branch Length when the Complement of *I* is not Empty

To generalize Equation (4), let *u* be a new internal node joining all the OTUs in $I=\{i_{1},i_{2},\ldots ,i_{P}\}$. Given any OTU $i \in I$, when $I^{\complement } \ne \emptyset$ we can sum the equality in Equation (8) for all the OTUs $k \notin I$:

18 $$\begin{aligned} \sum _{k \notin I} D_{ik} = (N-|I|) L_{iu} + \sum _{k \notin I} D_{uk}. \end{aligned}$$

Using the definition given in Equation (5) and substituting $D_{uk}$ with the expression in Equation (12), we obtain

19 $$\begin{aligned} R_{iI^{\complement }} = (N-|I|) L_{iu} + \sum _{k \notin I} \left( \frac{R_{Ik}}{|I|} - \frac{R_{II}}{|I|(|I|-1)} \right) . \end{aligned}$$

Now, we can use the definition given in Equation (6) and divide everything by $N-|I|$, obtaining

20 $$\begin{aligned} \frac{R_{iI^{\complement }}}{N-|I|} = L_{iu} + \frac{R_{II^{\complement }}}{|I|(N-|I|)} - \frac{R_{II}}{|I|(|I|-1)}, \end{aligned}$$

which, rearranging terms, finally yields

21 $$\begin{aligned} L_{iu} = \frac{R_{II}}{|I|(|I|-1)} + \frac{R_{iI^{\complement }}}{N-|I|} - \frac{R_{II^{\complement }}}{|I|(N-|I|)}, \end{aligned}$$

where $R_{II}$, $R_{iI^{\complement }},$ and $R_{II^{\complement }}$ are defined in Equations (11), (5), and (6), respectively.

#### Branch Length when the Complement of *I* is Empty

In case that all the remaining OTUs are clustered together in the same set *I* and, therefore, the set $I^{\complement }$ is empty, then the new internal node *u* joins all the remaining OTUs, and the distance between any OTU $i \in I$ and any other OTU $i' \in I$, $i' \ne i$, can be separated as follows:

22 $$\begin{aligned} D_{ii'} = L_{iu} + L_{i'u}. \end{aligned}$$

Summing this equality for all the OTUs $i' \in I$, $i' \ne i$, we obtain

23 $$\begin{aligned} R_{iI} = (|I|-1) L_{iu} + \sum _{i' \in I} L_{i'u} - L_{iu}, \end{aligned}$$

where $R_{iI}$ is the sum of distances between OTU $i \in I$ and all the other OTUs $i' \in I$, $i' \ne i$:

24 $$\begin{aligned} R_{iI} = \sum _{\begin{array}{c} i' \in I \\ i' \ne i \end{array}} D_{ii'}. \end{aligned}$$

Now, if we use Equation (10) for the sum of branch lengths of a starlike tree, we see that Equation (23) is equivalent to

25 $$\begin{aligned} R_{iI} = (|I|-2) L_{iu} + \frac{R_{II}}{|I|-1}, \end{aligned}$$

which, rearranging terms and dividing everything by $|I|-2$, can be finally expressed as follows:

26 $$\begin{aligned} L_{iu} = \frac{R_{iI}}{|I|-2} - \frac{R_{II}}{(|I|-1)(|I|-2)}. \end{aligned}$$

It is important to note here that both Equations (21) and (26) satisfy Equation (10) for the sum of branch lengths of a starlike tree.

In each iteration, all the OTUs in *I* are removed from the distance matrix, and the new node *u* is added. The procedure ends when all the remaining OTUs are clustered in the same set *I* and the set $I^{\complement }$ is empty. If there are no polytomies, this will happen for sure when the number of remaining OTUs is equal to three. In this case, Equation (26) reduces exactly to Equation (7). As a matter of fact, when there are no polytomies, the MFNJ algorithm reconstructs the same phylogenetic trees as the NJ algorithm.

To the best of our knowledge, there is only one method that deals with the ties in proximity problem: the extended neighbor-joining algorithm (Hong et al. 2021). Nevertheless, the formulas proposed in the extended neighbor-joining algorithm do not satisfy Equation (10) for the sum of branch lengths of a starlike tree, and the method is still limited because it can only join up to three OTUs to a new internal node. The MFNJ algorithm is more general than the extended neighbor-joining algorithm because Equations (12), (16), (21), and (26) can be used for any number of OTUs.

## Results

This section shows an example of the differences between the phylogenetic trees reconstructed by the NJ and the MFNJ algorithms using a specific distance matrix. In the case of the NJ algorithm, two possible phylogenetic trees are reconstructed. In the case of the MFNJ algorithm, only one phylogenetic tree is possible.

To do so, we used as input for both algorithms the matrix of distances given in Table 1. It is composed of the pairwise differences among mitochondrial DNA sequences of nine brown bears (*Ursus arctos* L.). We selected this case study because it had been previously used in one of the first articles that described the ties in proximity problem (Backeljau et al. 1996).

**Table 1:** Pairwise percentage differences among mitochondrial DNA sequences of nine brown bears (Randi et al. 1994)

	Abruzzo	Pyrenees	Kodiak	Captive-3	Captive-4	Captive-5	Grizzly	Polar-2
Pyrenees	1.3							
Kodiak	4.3	4.3						
Captive-3	4.3	4.3	0.7					
Captive-4	2.7	2.3	5.0	5.0				
Captive-5	3.0	3.0	1.3	1.3	3.7			
Grizzly	1.7	1.7	2.7	2.7	2.3	2.0		
Polar-2	2.0	2.0	3.0	3.0	2.7	2.3	0.3	
Black	8.7	8.0	10.0	10.0	10.0	8.7	9.0	9.4

**Fig. 4:**
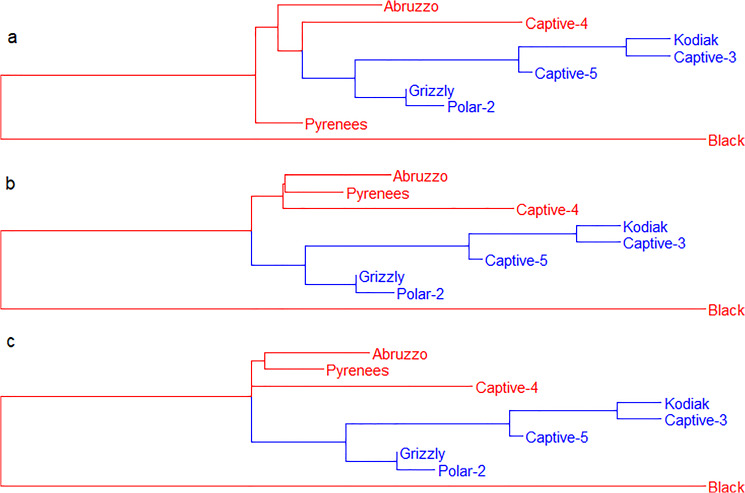
Phylogenetic trees obtained for the matrix of distances among bears given in Table 1. The trees have been plotted as rooted trees for convenience of comparison, where the longest branch has been placed at the root of each tree. At the fifth iteration of the algorithm, there is a tie between *Black*, *Captive-4,* and the subtree in blue. The bears in red are clustered during the last iterations of both the NJ and the MFNJ algorithms. **a, b** Two different dichotomic phylogenetic trees are possible when using the NJ algorithm. **c** A unique phylogenetic tree is possible when using the MFNJ algorithm, where a polytomy joining more than two subtrees can be observed (Color figure online)

After four iterations of the NJ algorithm, *Kodiak*, *Captive-3*, *Captive-5*, *Grizzly*, and *Polar-2* are clustered together in a subtree that we call *Subtree-4* (colored in blue in Fig. 4), and the other four bears remain nonclustered. At the fifth iteration of the algorithm, there is a tie between the pairs *Captive-4* and *Subtree-4*, and *Subtree-4* and *Black*, because their $S_{ij}$ values are equal and the smallest. Since the NJ algorithm cannot cluster three OTUs in a single step, two distinct phylogenetic trees are possible depending on the criterion used to break the tie. If *Captive-4* and *Subtree-4* are clustered first, then the phylogenetic tree in Fig. 4a is obtained. However, if *Subtree-4* and *Black* are clustered first, then the phylogenetic tree in Fig. 4b is obtained.

When the MFNJ algorithm is used with the same dataset, the first iterations are identical to the NJ algorithm, until the tie is found at the fifth iteration. Then, the MFNJ algorithm clusters *Captive-4*, *Subtree-4*, and *Black* at the same time forming a polytomy. Figure 4c shows the complete phylogenetic tree reconstructed by the MFNJ algorithm. This multifurcating tree is uniquely determined, what guarantees the reproducibility of any study on it.

## Conclusion

In this work, we propose a new method called the multifurcating neighbor-joining (MFNJ) algorithm, which is a generalization of the neighbor-joining (NJ) algorithm by Saitou and Nei (1987). The input of both algorithms is the same matrix of evolutionary distances among a set of taxa for which we want to reconstruct a phylogenetic tree. When there are no ties, the MFNJ algorithm gives the same results as the NJ algorithm. However, when ties exist, the advantage of the MFNJ algorithm is that it generates polytomic phylogenetic trees that do not depend on the order of taxa in the input matrix, whereas the NJ algorithm generates dichotomic phylogenetic trees that can be different depending on the order of the input taxa.

We have applied both the NJ and the MFNJ algorithms to the same real example composed of the pairwise differences among mitochondrial DNA sequences of nine brown bears. The NJ algorithm reconstructed two distinct dichotomic phylogenetic trees, depending on the order of the input data. Then, we have seen how this drawback can be easily avoided using the MFNJ algorithm, which reconstructs a unique polytomic phylogenetic tree for the same dataset.

We have shown the usability of the MFNJ algorithm with one example published in the literature. In future work, we plan to analyze the advantages of using the MFNJ algorithm on other public data presented in articles that have used the NJ algorithm. We also think that it is worthwhile to analyze bootstrap probabilities in case of nonunique dichotomic phylogenetic trees, since the existence of tied distances may have an important effect on these probabilities.
